# Oxidative Stress in Cardiorenal System

**DOI:** 10.3390/antiox13091126

**Published:** 2024-09-18

**Authors:** Carlos R. Tirapelli, Júlio C. Padovan

**Affiliations:** 1 Faculty of Pharmaceutical Sciences of Ribeirão Preto, University of São Paulo, Ribeirão Preto 14040-900, SP, Brazil; 2 Laboratory of Blood and Vascular Biology, The Rockefeller University, New York, NY 10065, USA; padovaj@rockefeller.edu

Reactive oxygen species (ROS) is a general term that describes free radicals [e.g., superoxide (O_2_^•−^) and hydroxyl radicals (^•^OH)] and non-radical molecules [e.g., hydrogen peroxide (H_2_O_2_)] that are naturally produced by cells as a result of metabolism. ROS are essential for cellular function as they activate intracellular signaling pathways sensitive to oxidation–reduction (redox) reactions (redox signaling pathways). The intracellular levels of ROS are controlled by enzymatic (e.g., catalase, superoxide dismutase, and glutathione reductase) and non-enzymatic [e.g., glutathione (GSH), ascorbic acid, and α-tocopherol] systems [1]. The overproduction of ROS or decreased antioxidant capacity disrupts the balance between ROS generation and elimination, leading to oxidative stress. In this context, ROS may cause cellular damage by directly reacting with cellular components (lipids, nucleic acids, carbohydrates, and proteins) or by promoting overactivation of redox-sensitive pathways [1,2]. Among all ROS, two species, O_2_^•−^ and H_2_O_2_, are essential to cell homeostasis. However, they are also responsible for cell damage in conditions that lead to the overproduction of ROS. The chemical stability and reactivity of these two species are different. The free-radical O_2_^•−^ is highly reactive and binds to macromolecules, promoting cellular damage, while H_2_O_2_, a more stable and less reactive molecule, is the main species involved in cell signaling through the activation of protein targets that are redox sensitive [1].

Excessive production of ROS leads to the development and progression of cardiorenal diseases. These molecules trigger intracellular responses associated with cardiorenal complications, such as inflammation, vascular/endothelial dysfunction, vasculature remodeling, apoptosis, cell migration, and expression of endothelial adhesion molecules. These processes are essential to the pathogenesis of hypertension, obesity, atherosclerosis, diabetes mellitus, and other conditions that affect the cardiorenal system [2]. Many enzymatic systems in the kidney, heart, and blood vessels contribute to the production of ROS. Among those, mitochondrial enzymes, nicotinamide adenine dinucleotide phosphate (NADPH) oxidase, and uncoupled endothelial nitric oxide (NO) synthase (eNOS) are particularly important. Of the ROS produced in such organs, O_2_^•−^ and H_2_O_2_ are notably relevant. These two species are associated with increases in intracellular calcium levels, decreases in NO bioavailability, lipid peroxidation, and activation of redox-signaling pathways [2,3]. Altogether, these responses lead to cardiorenal dysfunction and contribute to drug toxicity.

The Special Issue, Oxidative Stress in Cardiorenal System, comprises original contributions and review articles that bring new information into the relation between oxidative stress and cardiorenal diseases, focusing on cellular redox-signaling and how oxidative stress favors cardiovascular injury and drug toxicity. In the current Special Issue, Oliveira et al. (Contribution 1) evaluated whether perivascular adipose tissue (PVAT)-derived hydrogen sulfide (H_2_S) would counteract the vascular dysfunction in pregnancy hypertension, a condition that is characterized by overproduction of ROS and impaired endothelium-derived NO-induced vasodilation. Hydrogen sulfide is a vasorelaxant factor produced by the PVAT, endothelium, and smooth muscle cells [4]. The vasorelaxation induced by H_2_S may be diminished under pathophysiological conditions, and this response is associated with vascular dysfunction. The overproduction of ROS, which is seen during pre-eclampsia (pregnancy hypertension), abrogates the vasorelaxant action of H_2_S. Results from this original report revealed that oxidative stress associated with pre-eclampsia impairs vasodilation induced by endothelium-derived H_2_S. Interestingly, H_2_S produced by PVAT still maintained the anticontractile action of PVAT during endothelial dysfunction induced by pre-eclampsia. Additionally, vasodilation induced by PVAT-derived H_2_S was stimulated with the amino acid L-cysteine.

ROS play a role in the pathophysiology of many cardiorenal diseases, but they are also essential players in the toxic effects of some drugs used for therapeutic purposes. In this context, the contribution of da Silva et al. (Contribution 2) explored the mechanisms underlying the toxic effects of the chemotherapy drug 5-fluorouracil (5-FU) in the rat’s liver, kidney, and lung. The study provided evidence that 5-FU promoted pro-oxidative and pro-inflammatory responses, which were associated with histological and functional alterations. In the kidney, 5-FU increased lipoperoxidation, reduced the activities of catalase and superoxide dismutase, and decreased the levels of GSH. These responses occurred parallel to an increased renal accumulation of neutrophils and macrophages. The findings of da Silva et al. (Contribution 2) can be helpful in the search for cytoprotective agents that could abrogate or attenuate the adverse effects of 5-FU in the kidney.

Overproduction of ROS has a negative impact on tissue structure and function, so drugs that can prevent or inhibit oxidative stress are of interest. Statins, used to control cholesterol levels, emerge as potential candidates in this context as they exert pleiotropic effects, including endothelial protection and antioxidant effects [5]. Toghi et al. (Contribution 3) investigated the protective role of pravastatin against the vascular dysfunction induced by pregnancy hypertension. Using a rodent model of pre-eclampsia, they showed decreased production of NO, impaired vasorelaxation, and increased oxidative stress, responses observed along with the activation of matrix metalloproteinases (MMP)-2 in the placenta. Pravastatin restored NO levels and the endothelium-dependent relaxation in arteries from pregnant rats. The statin also attenuated oxidative stress-induced activation of MMP-2 in the placenta, suggesting that it may exert beneficial effects during pregnancy hypertension.

X-box binding protein 1 (XBP1) is a transcription factor whose activation may cause beneficial or harmful effects in the cardiorenal system. An oxidative imbalance in the cardiorenal system is seen during the aging process. Intracellular pathways activated by XBP1 are essential mediators of oxidative stress-induced cardiorenal dysfunction associated with aging [6]. Excessive activation of XBP1 during aging leads to mitochondrial dysfunction and ROS overproduction. The review by Zhang et al. (Contribution 4) details the mechanisms that link XBP1 and oxidative stress to the cardiorenal alterations induced by aging. XBP1 (via its role as a transcription factor) activates multiple redox-sensitive pathways that regulate the expression of a large variety of molecules related to mitochondrial (dys)function and morphology, the renin–angiotensin–aldosterone system (RAAS), and the immune and antioxidant systems.

In their review article, Padovan et al. (Contribution 5) discuss the mechanisms whereby ethanol (ethylic alcohol) induces vascular dysfunction, focusing on the role of ROS in such an effect. Ethanol-induced hypertension and cardiovascular toxicity is a multi-mediated event in which oxidative stress plays a central role. Excessive ethanol consumption up-regulates enzymatic systems that generate ROS and down-regulates enzymatic and non-enzymatic antioxidant systems. Their review also explores the relationship between neuroendocrine changes and the redox imbalance induced by ethanol in the vasculature. Ethanol induces the activation of the RAAS that promotes redox imbalance through the activation of angiotensin II type 1 receptors and mineralocorticoid receptors. In this scenario, ROS intermediate the intracellular responses triggered by the RAAS.

Mladenov et al. (Contribution 6) provided an update on the mechanisms that regulate health, disease, and aging redox status. The review describes the main cellular signaling pathways that counteract reductive and oxidative stress. Information on the function of food components with antioxidant properties in cellular homeostasis is also provided. The participation of the hormones irisin and melatonin in redox balance and the correlations between the deviation from ideal redox state and inflammation, aging, and autoimmune responses are also discussed. Special attention is given to redox imbalance in the vasculature, kidney, liver, and brain.

Along with vasoconstriction and hypoperfusion, the generation of ROS is essential to the occurrence of contrast-associated acute kidney injury (CA-AKI) in patients with ST-elevation myocardial infarction (STEMI). The hypothesis study of Arrivi et al. (Contribution 7) speculated that the occurrence of CA-AKI in STEMI patients who undergo primary percutaneous coronary intervention may be counteracted by intravenous infusion of glutathione and ascorbic acid. A multicenter research protocol was used to evaluate the hypothesis. A double-blind, randomized, placebo-controlled trial design was used in the research protocol.

The Special Issue provided scientifically innovative publications, stimulating broader discussion in the field. The scientific publications (three original contributions, three review articles, and one hypothesis study) highlight the deleterious effects of oxidative stress in the cardiorenal system, detailing the molecular mechanisms underlying the harmful effects of ROS and providing therapeutic/natural approaches to mitigate the prejudicial action of ROS. We want to thank the contributors for their time, effort, and expertise in preparing their articles. The original studies and review articles published in this issue provide new insights into the participation of ROS in drug toxicity and pathogenesis/treatment of cardiorenal diseases, which we hope the readers will find both informative and stimulating.
